# High-Performance Cobalt-Free SrFe_1−x_Ce_x_O_3−δ_ Cathode Enabled by Ce-Driven Defect Engineering for IT-SOFCs

**DOI:** 10.3390/ma19194245

**Published:** 2026-10-07

**Authors:** Packiaraj Rajagopal, Mahitha Rajasekaran, Sivaganesh Dhanushkodi, Ainur Aimakhanova, Mukhammed Kenzhebek, Aliya Baratova, Marzhan Kubenova, Lukman Ahmed Omeiza, Asset Kabyshev

**Affiliations:** 1 Institute of Physical and Technical Sciences, L.N. Gumilyov Eurasian National University, Astana 010008, Kazakhstan; aynur.aymakhanova.86@mail.ru (A.A.); mukhammed.kenzhebek@nu.edu.kz (M.K.); baratova_aa@enu.kz (A.B.); kubenova_mm_1@enu.kz (M.K.);; 2 Department of Physics, School of Advanced Sciences, Kalasalingam Academy of Research and Education, Virudhunagar 626126, India; r.mahitha@klu.ac.in; 3 Research Group for Advanced Materials and Sustainable Catalysis, State Key Laboratory Breeding Base of Green Chemistry-Synthesis Technology, College of Chemical Engineering, Zhejiang University of Technology, Hangzhou 310032, China; ganesh.siva650@gmail.com; 4 Anhui International Exchange and Cooperation Base, Qing Yang Institute for Industrial Minerals, Qingyang 242804, China; 5 Faculty of Integrated Technologies, Universiti Brunei Darussalam, Gadong BE1410, Brunei

**Keywords:** SrFeO_3−δ_, cerium substitution, cobalt-free perovskite, oxygen defects, mixed ionic-electronic conduction, oxygen reduction reaction, solid oxide fuel cells

## Abstract

**Highlights:**

- Cobalt-free SrFe_1−x_Ce_x_O_3−δ_ (x = 0, 0.05, 0.10, 0.15 and 0.20) cathodes were systematically developed for IT-SOFCs.
- Ce substitution effectively tuned crystal structure, defect chemistry, and Fe redox states.
- SFCO-3 exhibited the optimum balance of conductivity and electrochemical activity.
- Ce-driven defect engineering enhanced oxygen transport and reduced cathodic polarization.
- The optimized cathode demonstrated promising single-cell performance from 600 to 800 °C.

**Abstract:**

Cobalt-free SrFeO_3−δ_ is an attractive mixed ionic-electronic conducting (MIEC) oxygen-electrode platform for intermediate-temperature solid oxide fuel cells (IT-SOFCs), but its electrochemical response is strongly coupled to Fe valence, oxygen non-stoichiometry, and structural phase stability. Here, B-site Ce-substituted SrFe_1−x_Ce_x_O_3−δ_ (x = 0, 0.05, 0.10, 0.15, and 0.20) was prepared by a solid-state route to establish a composition-structure-defect-transport relationship. X-ray diffraction and Rietveld refinement show an initial expansion of the perovskite lattice after Ce introduction, followed by a lattice-parameter plateau and weak CeO_2_/Sr_3_Fe_2_O_7−δ_ segregation at higher Ce contents. Electron microscopy confirms an interconnected granular morphology, while HRTEM/SAED of SrFe_0.85_Ce_0.15_O_3−δ_ verifies well-crystallized perovskite domains. XPS reveals a Ce-dependent redistribution of the Fe^3+^/Fe^4+^ and surface oxygen environments, with the x = 0.15 composition displaying the most favorable defect-associated oxygen response. Electrical conductivity follows a non-monotonic composition dependence and is maximized at x = 0.15, indicating an optimum balance between defect formation and continuity of the Fe-O-Fe transport network. A single cell employing SrFe_0.85_Ce_0.15_O_3−δ_ as the oxygen electrode delivers a peak power density of 0.53 W cm^−2^ at 800 °C. The corresponding polarization resistance decreases from 0.47 Ω at 600 °C to 0.36 Ω at 800 °C, confirming strongly activated oxygen-electrode kinetics. These results identify controlled B-site Ce substitution as an effective route for tuning the electronic/defect chemistry of cobalt-free SrFeO_3−δ_ cathodes.

## 1. Introduction

Lowering the operating temperature of solid oxide fuel cells (SOFCs) from the conventional high-temperature regime to approximately 600–800 °C is essential for suppressing interfacial reactions, electrode coarsening, thermomechanical degradation, and sealing-related failures. At lower operating temperatures, cathodic polarization rises as oxygen adsorption and dissociation, surface exchange, electron transfer, and oxide-ion incorporation slow down. Consequently, the oxygen electrode remains one of the major kinetic bottlenecks in intermediate-temperature SOFCs (IT-SOFCs), and current materials design seeks to combine high electronic conductivity, mobile oxygen defects, rapid surface exchange, phase stability, and electrolyte compatibility within a single electrode framework [1,2].

La_1−x_Sr_x_MnO_3−δ_ (LSM) is the classical SOFC cathode because of its electronic conductivity, chemical robustness, and compatibility with yttria-stabilized zirconia. However, its negligible bulk oxide-ion conductivity confines the oxygen reduction reaction (ORR) largely to the gas/electrode/electrolyte triple-phase boundary, producing substantial polarization at reduced temperature. La_0.6_Sr_0.4_Co_0.2_Fe_0.8_O_3−δ_ (LSCF) overcomes this limitation through mixed ionic-electronic conduction (MIEC), extending the reaction zone beyond the conventional triple-phase boundary. Despite its high activity, LSCF remains vulnerable to Sr surface segregation, interfacial reactions, contaminant poisoning, and the relatively large thermal expansion associated with Co-containing lattices [1,2]. In order to separate ORR activity from degradation, these constraints have prompted the development of composite electrodes, infiltration, surface decorating, exsolution, hetero interface engineering, and cation-defect techniques.

A second state-of-the-art approach is offered by layered and double-perovskite oxygen electrodes. Because cation ordering and anisotropic oxygen-defect sublattices reduce oxygen-transport barriers, systems generated from LnBaCo_2_O_5+δ_ can display fast oxygen diffusion and surface exchange. The activity and CO_2_ tolerance of these double perovskites have been further enhanced by medium-entropy engineering [3]. Similarly, mixed-conducting PrNi_0.5_Co_0.5_O_3−δ_ shows how oxygen non-stoichiometry and coupled redox states can maintain high electrode activity while simultaneously demonstrating that thermal expansion and electrolyte compatibility are still crucial at the cell level [4]. However, the persistence of Co in many high-performance formulations encourages the development of less expensive and possibly more thermochemically compatible cobalt-free substitutes.

In this context, SrFeO_3-δ_ is particularly appealing due to its Fe-O framework, which facilitates mixed Fe^3+^/Fe^4+^ chemistry and significant oxygen nonstoichiometry. The same defect flexibility that enables mixed conduction also makes the material highly sensitive to oxygen loss: changes in δ alter Fe coordination, lattice symmetry, carrier concentration, and oxygen mobility. Undoped SrFeO_3-δ_ has nevertheless demonstrated useful SOFC activity, including a peak power density of 202 mW cm^−2^ at 800 °C in an electrolyte-supported symmetric cell [5].

B-site substitution has therefore become an effective means of stabilizing the lattice and regulating transport. Si substitution improved the conductivity and electrode resistance by modifying oxygen-vacancy ordering [6]; W substitution stabilized the cubic phase and enabled 931 mW cm^−2^ in an LSGM-supported symmetric cell [7]; Ti substitution produced an initial ASR of 0.03 Ω cm^2^ in O_2_ and 509 mW cm^−2^ at 800 °C [8]. A-site Pr substitution has also been shown to reduce thermal expansion while retaining favorable conductivity and electrochemical performance [9]. These studies establish that SrFeO_3−δ_ performance is controlled by an optimum balance between oxygen-defect chemistry and preservation of the electronically connected Fe-O-Fe network. More broadly, recent work on ferrite-based functional ceramics has demonstrated that dopant chemistry and sintering-driven microstructural evolution can strongly influence functional performance. In co-doped MnZn ferrites, optimized dopant combinations promoted grain refinement and increased grain-boundary resistivity, thereby suppressing high-frequency power loss; notably, the optimum dopant concentrations under co-doping conditions differed from those identified in single-doped systems [10].

Cerium offers an interesting lever for adjusting this equilibrium since Ce^4+^/Ce^3+^ redox chemistry and strong Ce-O bonding can change oxygen-defect thermodynamics while also affecting transition-metal valence. Ce substitution has improved structural stability and ORR activity in SrCoO_3−δ_ [11]. However, Fe-site Ce substitution in the chemically analogous BaFe_1−x_Ce_x_O_3−δ_ system has been shown to modify oxygen-ion and electronic transport in opposite directions. Increasing Ce content may improve oxide-ion mobility while reducing electron and hole mobility, as Ce substitution interrupts the Fe-based electronic transport network [12]. Excessive B-site substitution in SrFeO_3-δ_ can disrupt Fe-O-Fe pathways and cause secondary-phase segregation, making this a crucial trade-off. Although A-site Ce-containing SrFeO_3-δ_ has been investigated for transport and symmetric-cell applications [13,14,15], direct systematic evaluation of Ce on the Fe site remains comparatively limited.

Here, SrFe_1−x_Ce_x_O_3−δ_ (x = 0, 0.05, 0.10, 0.15, and 0.20) is investigated as a cobalt-free oxygen-electrode series with emphasis on B-site Ce-driven structure-defect-transport coupling. XRD/Rietveld refinement, electron microscopy, HRTEM/SAED, XPS, particle-size analysis, and temperature-dependent conductivity are combined to identify the composition at which Ce-induced lattice/defect modification remains compatible with efficient electronic transport. The selected composition was subsequently evaluated in a single SOFC using current-voltage, power-density, and electrochemical impedance measurements. The resulting analysis is used to connect Ce-induced structural modification with Fe redox chemistry, oxygen-defect response, charge transport, and ORR polarization.

## 2. Materials and Methods

### 2.1. Preparation of SrFe_1−x_Ce_x_O_3−δ_

**Figure 1:**
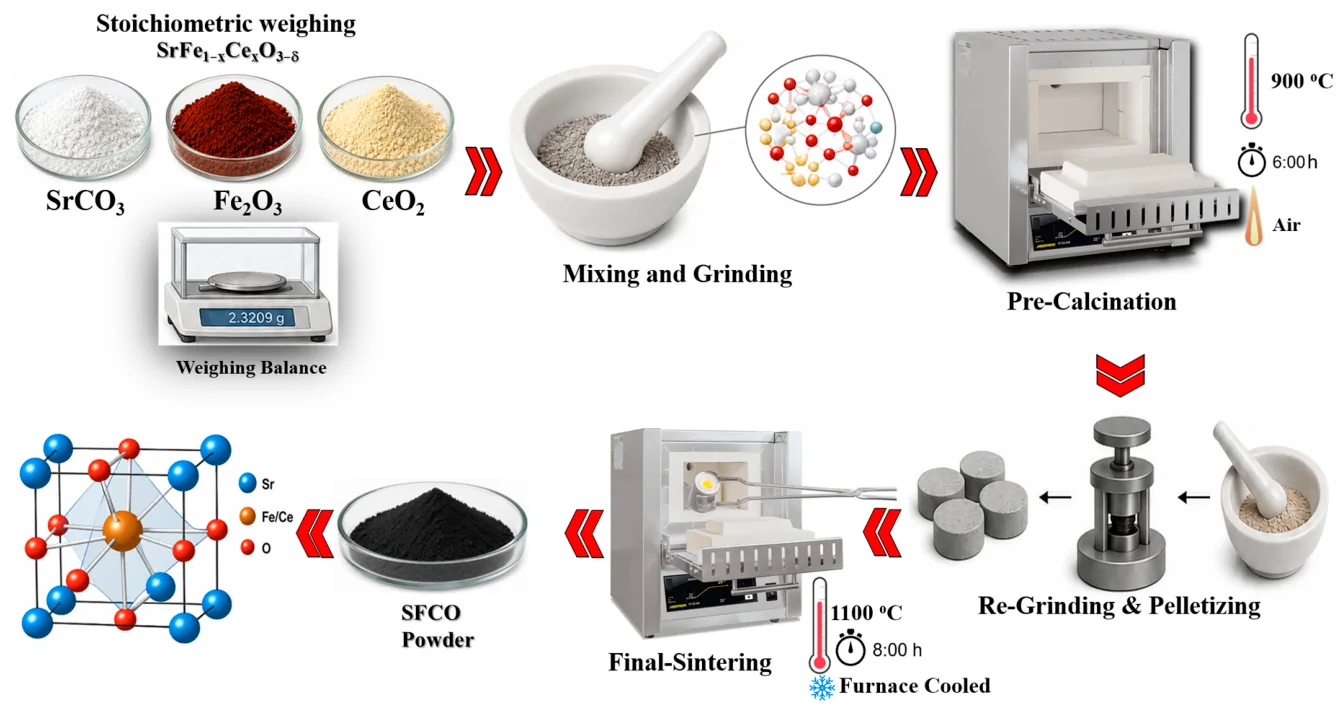
Schematic illustration of the solid-state synthesis of SFO and Ce-substituted SFCO-1, SFCO-2, SFCO-3, and SFCO-4 perovskites.

Pristine SrFeO_3−δ_ and Ce-substituted SrFe_1−x_Ce_x_O_3−δ_ (x = 0.05, 0.10, 0.15, and 0.20) were synthesized by a conventional solid-state reaction route, as illustrated in Figure 1. High-purity SrCO_3_, Fe_2_O_3_, and CeO_2_ (99.99%) were weighed in stoichiometric amounts calculated for each nominal composition. The precursors were then wet-ground with approximately 10 mL of ethanol for 4 h to ensure thorough mixing. The resulting mixtures were dried overnight at 100 °C, followed by calcination in air at 900 °C for 6 h using a heating rate of 5 °C min^−1^. After cooling, the calcined powders were reground for 30 min, loaded into a 32 mm stainless-steel die, and compacted under a uniaxial load of 90 kN using a hydraulic press. Final sintering was carried out in air at 1100 °C for 8 h with a heating rate of 3 °C min^−1^, followed by furnace cooling. Heat-treated pellets or reground powders were subsequently used for structural, physicochemical, electrical, and electrochemical characterization. For clarity, the compositions are designated SFO (SrFeO_3-δ_), SFCO-1 (SrFe_0.95_Ce_0.05_O_3−δ_), SFCO-2 (SrFe_0.90_Ce_0.10_O_3−δ_), SFCO-3 (SrFe_0.85_Ce_0.15_O_3−δ_), and SFCO-4 (SrFe_0.80_Ce_0.20_O_3−δ_) throughout the manuscript.

### 2.2. Characterization

Structural and phase evolution was examined by powder X-ray diffraction (XRD; Bruker D8 Advance) using Cu Kα radiation (λ = 1.54 Å) with a Ni filter, operated at an X-ray generator voltage of 40 kV and a current of 25 mA, with an SSD160 detector. The diffraction profiles were analyzed by Rietveld refinement using [JANA2006] software to determine the average lattice parameter, unit-cell volume, and profile reliability indices. Surface morphology and elemental distribution were examined using a Hitachi TM3030 scanning electron microscope (Hitachi, Tokyo, Japan) coupled with a (Bruker XFlash MINSVE energy-dispersive X-ray spectroscopy (EDX, Bruker Nano GmbH, Berlin, Germany)) microanalysis system at an accelerating voltage of 15 kV. The local crystallographic structure of the optimized SFCO-3 composition was investigated by high-resolution transmission electron microscopy and selected-area electron diffraction (HRTEM/SAED) using a (JEOL JEM-2100, JEOL Ltd., Tokyo, Japan) instrument. Surface chemical states and oxygen-related species were investigated by X-ray photoelectron spectroscopy (XPS: Thermo Scientific NEXSA, Thermo Fisher Scientific, East Grinstead, UK). Particle-size distributions of the sintered powders were measured using a Mastersizer 2000 particle-size analyzer (Malvern Instruments, Malvern, UK).

For electrical conductivity measurements, pellets were prepared separately from all five compositions: SFO, SFCO-1, SFCO-2, SFCO-3, and SFCO-4. For each composition, 6 g of powder was granulated with approximately five drops of 6 wt.% polyvinyl alcohol (PVA) solution and compacted under a load of 90 kN using a hydraulic press (TG1220MA, 20-ton capacity; ARMADA, Moscow, Russia). The green pellets were sintered at 1100 °C for 2 h. Bulk density and apparent porosity were determined for each composition by the Archimedes method before electrical conductivity measurements.

### 2.3. Electrical Conductivity

Temperature-dependent electrical conductivity was measured on sintered pellets of all compositions over the investigated temperature range. Van der Pauw four-probe DC method was used with a Keithley 6221 current source (100 mA) and a Keithley nanovoltmeter, Keithley Instruments, Solon, OH, USA. Four silver wire (t = 250 micron) contacts were attached to the pellet using silver paste. The samples were dried and heated in a furnace at 800 °C for 30 min to improve adhesion of the silver contacts before conductivity measurements were performed. The conductivity data were compared across the series to identify the composition providing the most favorable bulk charge transport.

### 2.4. Single-Cell Fabrication and Electrochemical Testing

Commercial 8 mol% Y_2_O_3_-stabilized ZrO_2_ (8YSZ) electrolyte discs with a diameter of 20 mm and a nominal thickness of 900 μm were purchased from E-Chem Supplies. The actual electrolyte thickness was approximately 912 μm, as determined from cross-sectional FESEM analysis. The fuel-electrode paste was prepared by mechanically mixing NiO powder (99.9%, Inframat, Manchester, CT, USA) and 8YSZ powder (Tosoh Zirconia TZ-8Y, Tosoh Corporation, Tokyo, Japan) in a weight ratio of 62.5:37.5, together with ethyl cellulose (Loba Chemie, Mumbai, India) as a binder and terpineol (80%, Loba Chemie) as the organic vehicle. The mixture was thoroughly ground with a mortar and pestle until a homogeneous paste with a viscosity that was suitable for screen printing was achieved. The NiO-8YSZ paste was screen-printed onto one side of the 8YSZ electrolyte over an active area of 1 cm^2^ and subsequently sintered at 1300 °C for 2 h to form the half-cell. The oxygen-electrode paste was prepared in a similar manner using 5 g of calcined SFCO-3 powder, ethyl cellulose, and a few drops of terpineol. The resulting paste was screen-printed onto the opposite side of the electrolyte over an active area of 1 cm^2^ and sintered in air at 1100 °C for 2 h. The resulting asymmetric cell had the configuration NiO-8YSZ (34 μm)||8YSZ (912 μm)||SFCO-3 (27 μm).

The electrochemical performance of the fabricated button cells was evaluated using a fuel-cell test station (Fiaxell, Lausanne, Switzerland). Current-voltage (I-V), power-density, and related electrochemical measurements were carried out at 600, 700, and 800 °C using a Squidstat^TM^ Penta potentiostat/galvanostat/FRA (Admiral Instruments, Tempe, AZ, USA). A Manson HCS-3202 switching-mode DC power supply (1–36 V DC, 0–10 A; Manson Engineering Industrial Ltd., Kwai Chung, Hong Kong) was used as an auxiliary DC power source during testing. Electrochemical impedance spectroscopy (EIS) measurements were performed using a CS2350 bipotentiostat (Corrtest Instruments, Wuhan, China) under open-circuit voltage (OCV) conditions with an AC perturbation amplitude of 10 mV over a frequency range of 1 MHz to 0.1 Hz. The high-frequency intercept was treated as the series/ohmic resistance (R_s_). For the single dominant electrode arc used in the present fitting, the arc resistance was denoted R_ct_ and is equivalent to the polarization resistance R_p_; the total resistance is R_total_ = R_s_ + R_p_.

## 3. Results and Discussion

### 3.1. Powder X-Ray Diffraction Analysis

**Figure 2:**
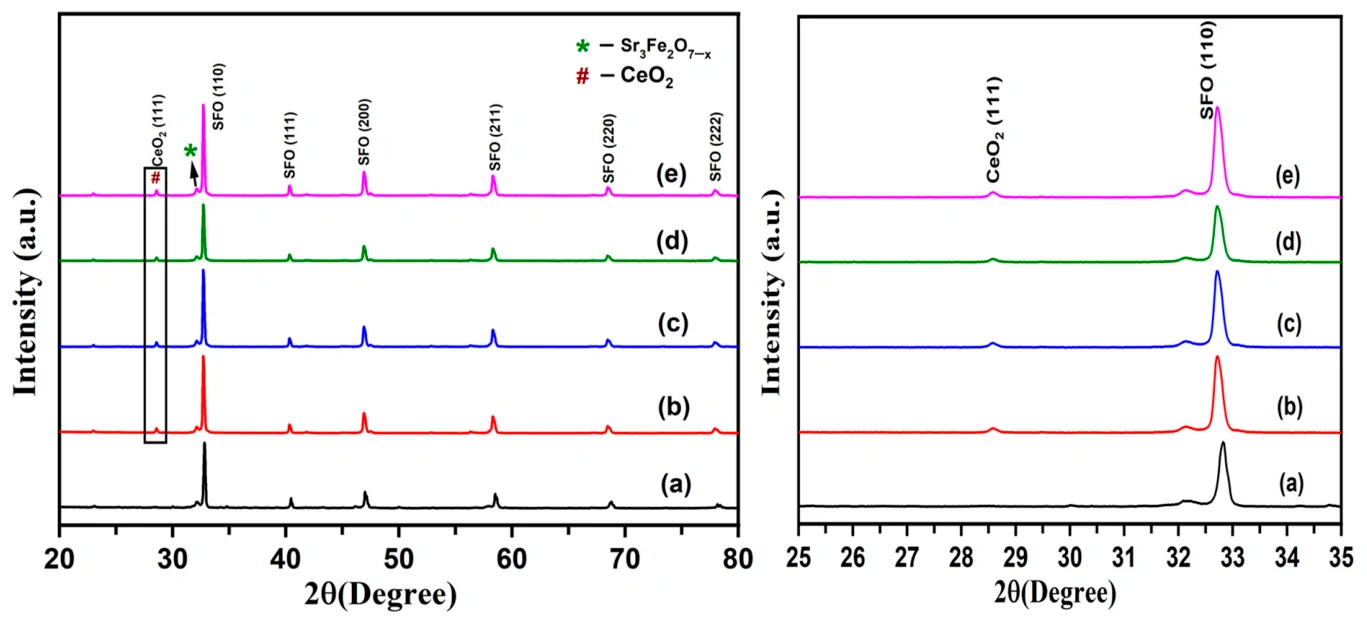
XRD patterns and enlarged CeO_2_ (111) and SFO (110) reflections of (a) SFO, (b) SFCO-1, (c) SFCO-2, (d) SFCO-3and (e) SFCO-4.

The powder X-ray diffraction patterns of SFO, SFCO-1, SFCO-2, SFCO-3, and SFCO-4 are dominated by reflections of the cubic SrFeO_3-δ_ perovskite framework (space group Pm-3m), indexed to the (110), (111), (200), (211), (220), and (222) planes, indicating that the parent perovskite remains the principal crystalline phase throughout the series (Figure 2). The enlarged CeO_2_ (111) and SFO (110) reflection shifts toward lower (2θ) following Ce incorporation, indicating an increase in interplanar spacing and a measurable perturbation of the host lattice. Such expansion is consistent with partial Ce incorporation into the B-site of the SrFeO_3−δ_ perovskite lattice, as six-coordinate Ce^4+^ has a substantially larger ionic radius than Fe^4+^/Fe^3+^, which can locally expand and strain the Fe/Ce-O framework [12].

Analogous B-site Ce substitution in BaFe_1−x_Ce_x_O_3−δ_ has likewise been shown to couple lattice dimensions with Ce redox chemistry and oxygen/electronic defect responses [12,16]. Notably, the structural evolution is not strictly monotonic with Ce content. A weak CeO_2_ (111) reflection around 28–29° indicates that the nominal Ce content is not accommodated exclusively within the perovskite lattice, while at the highest substitution level an additional weak Sr_3_Fe_2_O_7−δ_-related reflection suggests incipient redistribution toward a layered Ruddlesden-Popper-type ferrate environment. These results suggest that Ce initially incorporates, at least partially, into the SrFeO_3−δ_ perovskite lattice, whereas further increases in nominal Ce content approach the accommodation limit of the perovskite under the present synthesis conditions.

**Figure 3:**
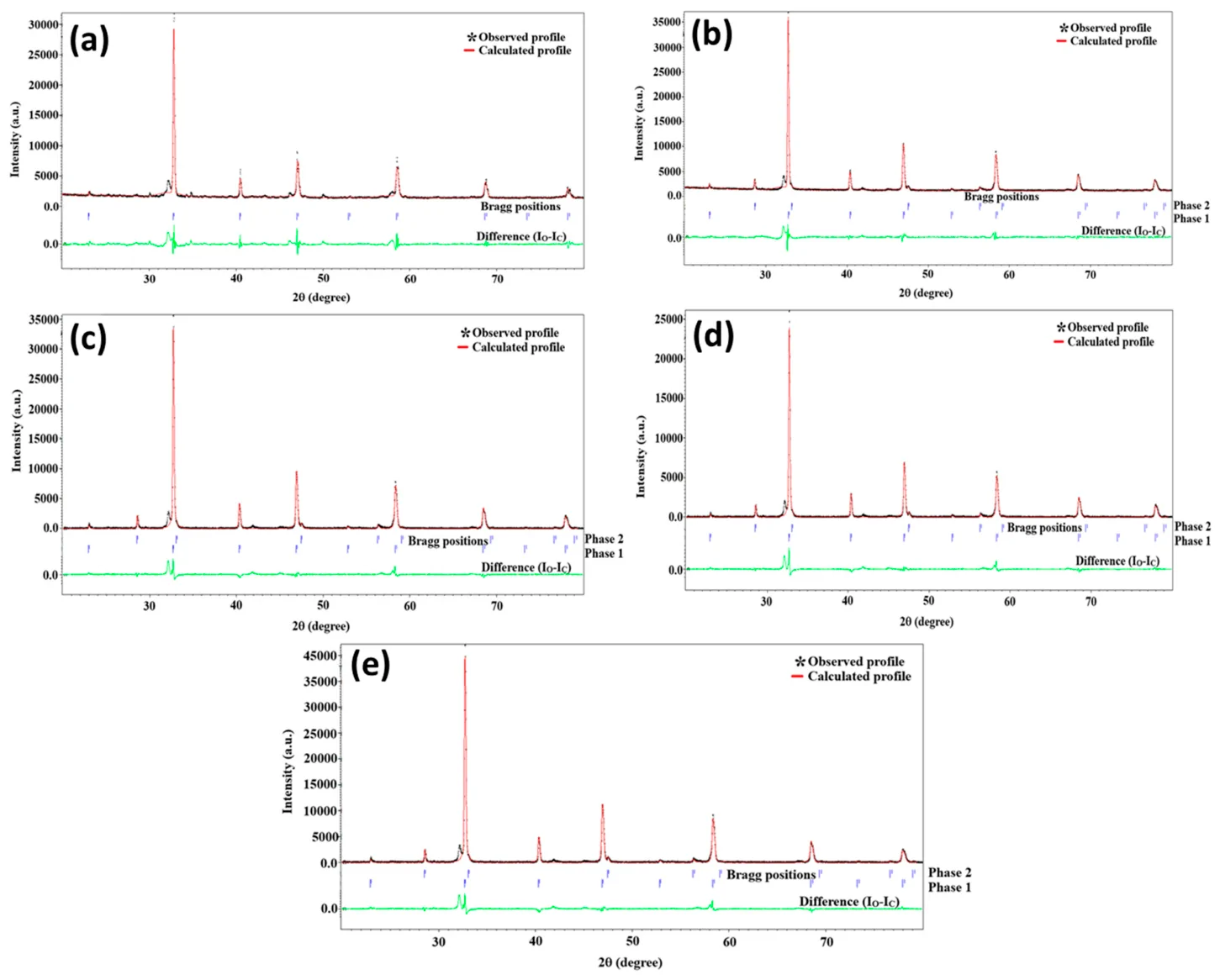
Rietveld refinement profiles of (**a**) SFO, (**b**) SFCO-1, (**c**) SFCO-2, (**d**) SFCO-3 and (**e**) SFCO-4.

Rietveld refinement using JANA2006 [17,18] quantitatively supports this interpretation (Figure 3 and Table 1). The refined cubic lattice parameter increases from 3.8617(13) Å for pristine SrFeO_3−δ_ to 3.8758(8) Å for (x = 0.05), with a corresponding increase in unit-cell volume from 57.59(2) to 58.22(1) Å^3^. In contrast, further Ce addition produces essentially no statistically meaningful expansion, with lattice parameters of 3.8745(15), 3.8746(15), and 3.8745(15) Å and unit-cell volumes of 58.16(2), 58.17(2), and 58.16(2) Å^3^ for (x = 0.10, 0.15, and 0.20, respectively. The series therefore departs from a simple Vegard-type evolution and instead exhibits pronounced initial lattice relaxation followed by structural saturation. This plateau, together with weak CeO_2_ segregation and the Sr_3_Fe_2_O_7−δ_ contribution at high (x) is consistent with increasing local strain and partial phase redistribution once the perovskite reaches its effective Ce-accommodation limit under the present synthesis conditions. Similar non-monotonic behavior has been reported for other B-site-modified SrFeO_3−δ_ systems, where moderate substitution can stabilize or tune the perovskite lattice, whereas excessive substitution promotes secondary-phase formation or disruption of the Fe-O framework [6,7,8,19]. The refinement quality remains comparable across all compositions, with (R_obs_ = 2.34–2.85), (R_p_ = 5.03–5.26), and GOF = 3.01–3.05, indicating satisfactory agreement between the observed and calculated profiles while preserving the same average structural model across the series.

### 3.2. Microstructure, Elemental Distribution, Particle Size Analysis and Density Measurement

The FESEM images (Supplementary Figure S1) show that all compositions consist of irregular submicrometric grains assembled into larger agglomerates, with open intergranular voids retained after high-temperature processing. At higher magnification, adjacent grains exhibit partial necking rather than complete densification. This morphology is advantageous for an oxygen electrode because particle-particle contacts provide an electronically continuous backbone, while the residual pore network preserves gas access to the reactive surface. Across the Ce series, no abrupt morphological reconstruction is observed, indicating that the principal effect of Ce is exerted through lattice/defect chemistry rather than gross particle-shape transformation.

EDS analysis further confirms the elemental constitution and spatial distribution of the SrFe_1−x_Ce_x_O_3−δ_ series (Supplementary Figure S1). The spectrum of pristine SFO contains only Sr, Fe, and O, whereas an additional Ce signal appears in all Ce-substituted compositions, consistent with successful introduction of Ce into the material. Elemental maps show that Sr, Fe, and O are broadly distributed throughout the particles, while Ce is dispersed across the doped samples without evidence of pronounced micrometer-scale segregation. Minor local Ce-enriched regions are nevertheless visible in some compositions, which is consistent with the weak CeO_2_ contribution detected by XRD at higher Ce contents. These results therefore support preservation of the Sr-Fe-O matrix after Ce addition while indicating that Ce is predominantly distributed within the microstructure, with limited local compositional heterogeneity at elevated substitution levels. Because EDS provides elemental rather than valence or site-specific information, the maps alone do not establish the exact crystallographic position or oxidation state of Ce.

**Figure 4:**
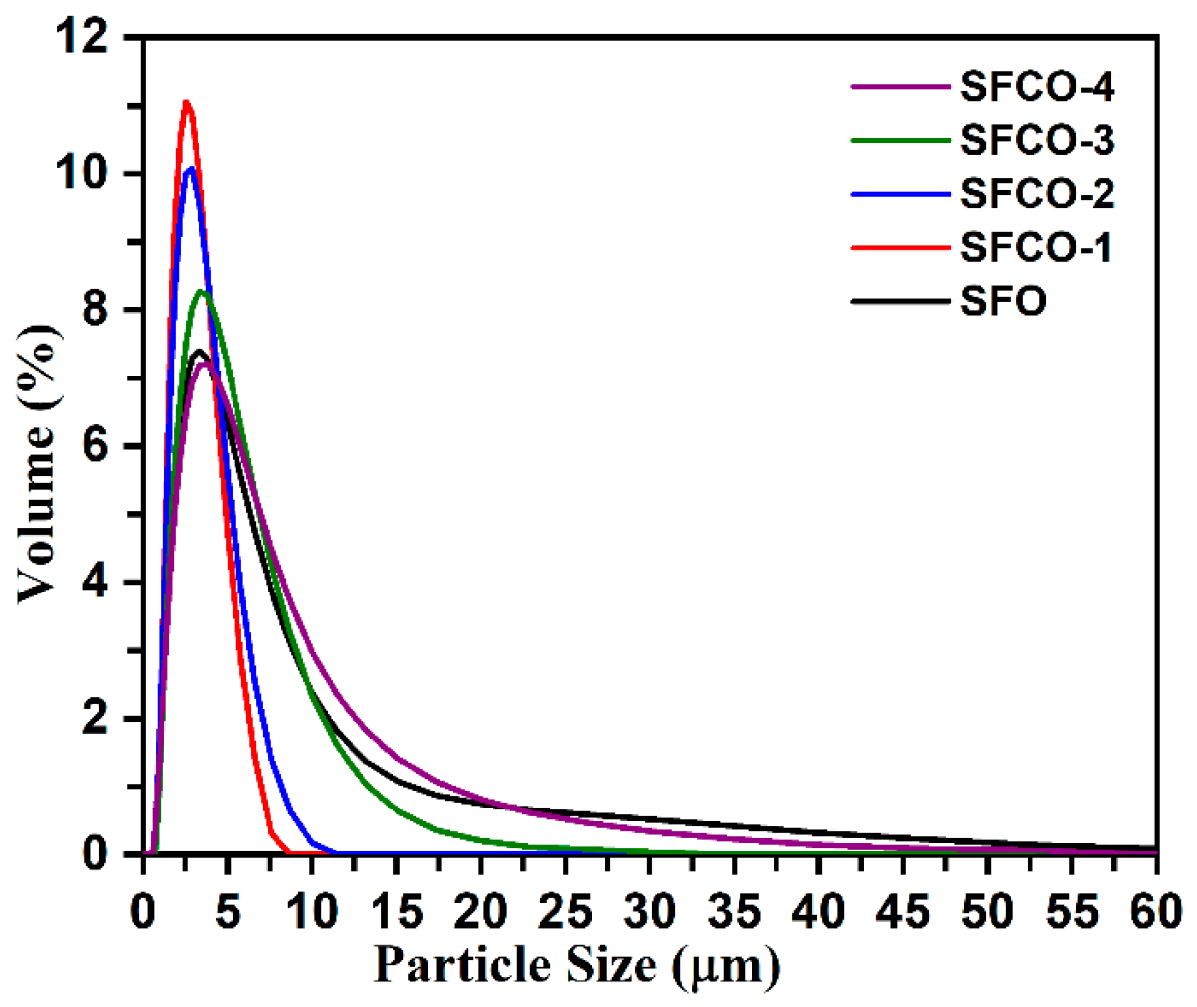
Volume-weighted particle-size distributions of sintered SFO, SFCO-1, SFCO-2, SFCO-3, and SFCO-4 powders plotted on common axes.

The volume-weighted particle-size distributions of the sintered powders are compared on common axes in Figure 4. The distribution maximum shifts from approximately 3.31 µm for SFO to 2.51 µm for SFCO-1, then moves toward larger sizes with further Ce substitution, reaching 3.80 µm for SFCO-4. SFCO-1 has the narrowest distribution, with D_10_, D_50_, and D_90_ values of 1.451, 2.720, and 4.930 µm, respectively, and a span of 1.279. The distribution subsequently broadens: the span increases to 1.432 for SFCO-2, 1.868 for SFCO-3, and 2.394 for SFCO-4. SFCO-4 also has the largest D_50_ (4.001 µm) and a pronounced tail toward larger sizes, consistent with a greater contribution from agglomerated particles. These laser-diffraction measurements describe dispersed powder particles and agglomerates rather than coherent XRD crystallites; they should therefore be interpreted alongside, rather than equated with, the FESEM observations.

The SFO, SFCO-1, SFCO-2, SFCO-3, and SFCO-4 pellets were sintered at 1100 °C for 2 h before bulk density and apparent porosity were measured by the Archimedes method. The reported bulk densities for SFO, SFCO-1, SFCO-2, SFCO-3, and SFCO-4 are 5.36, 6.91, 8.55, 13.22, and 6.45 g cm^−3^, respectively. Their apparent porosities were 6%, 8%, 14%, 22%, and 10%, respectively. SFCO-3 exhibited the highest apparent porosity.

### 3.3. HRTEM and SAED Analysis

**Figure 5:**
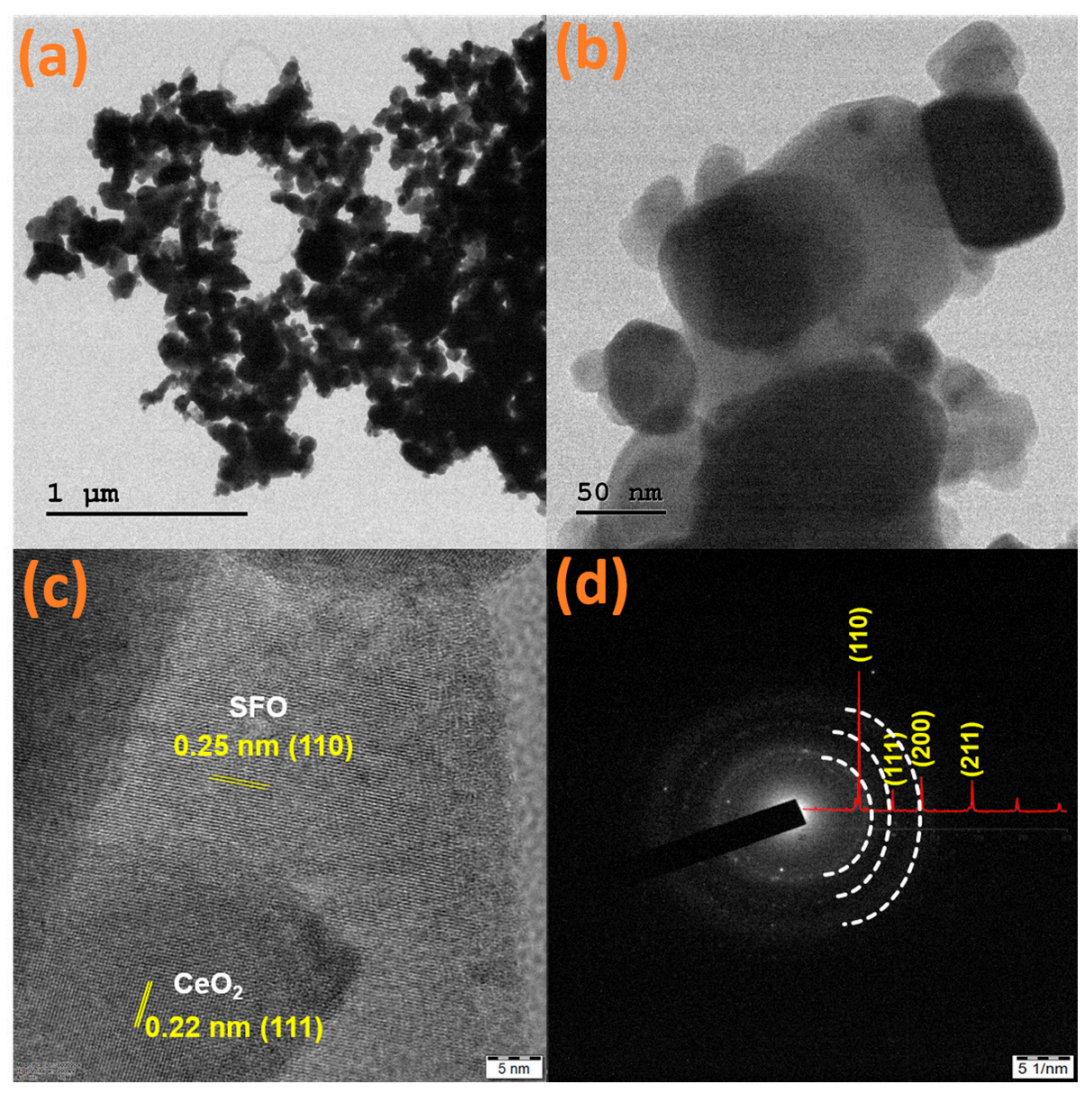
(**a**–**c**) HRTEM images of SFCO-3 at different magnifications and (**d**) the corresponding selected-area electron diffraction (SAED) pattern. The dotted circles highlight the diffraction rings.

HRTEM analysis of the optimized SFCO-3 composition reveals well-resolved lattice fringes, confirming a high degree of local crystallinity, as shown in Figure 5. The low-magnification HRTEM images (Figure 5a,b) show the overall morphology and aggregation of the SFCO-3 particles. These images provide an overview of the particle arrangement before the lattice fringes are examined at higher magnification. The measured interplanar spacing of approximately 0.254 nm can be indexed to the (110) plane of the SrFeO_3−δ_ perovskite structure and is consistent with the lattice parameter obtained from Rietveld refinement. The corresponding SAED pattern exhibits distinct spot-decorated diffraction rings, characteristic of a polycrystalline material composed of well-ordered crystalline domains. The absence of a pronounced diffuse halo further indicates that the examined region contains little amorphous contribution. These observations corroborate the XRD results and demonstrate that the x = 0.15 composition retains well-developed perovskite lattice order at the nanoscale despite Ce-induced structural perturbation.

### 3.4. X-Ray Photoelectron Spectroscopy (XPS) Analysis

**Figure 6:**
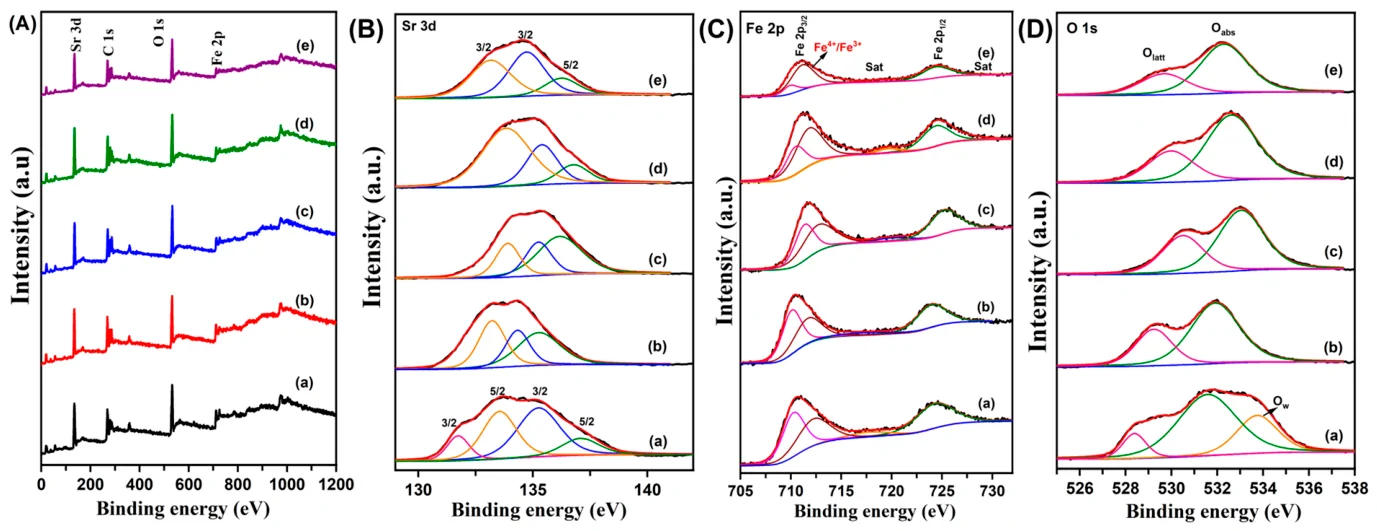
XPS spectra of SFO, SFCO-1, SFCO-2, SFCO-3, and SFCO-4: (**A**) survey spectra and high-resolution (**B**) Sr 3d, (**C**) Fe 2p, and (**D**) O 1s spectra. In each panel, traces (a–e) correspond to SFO through SFCO-4, respectively.

The survey XPS spectra (Figure 6A) confirm Sr, Fe, and O as the dominant surface constituents, while the Ce-related signal emerges after substitution and increases with nominal Ce content. The weak C 1s contribution is attributed to adventitious surface carbon. The persistence of the Sr-Fe-O core-level features across the series indicates that Ce addition modifies the chemical state of the ferrite surface without replacing the parent oxide chemistry with a new dominant phase.

The Sr 3d spectra (Figure 6B) retain the characteristic Sr 3d_5/2_-Sr 3d_3/2_ doublet associated predominantly with Sr^2+^ in the perovskite A-site. A higher-binding-energy contribution is consistent with surface Sr-containing hydroxyl/carbonate-like species commonly observed after ambient exposure of Sr-based perovskites. The overall similarity of the Sr envelopes across the series indicates that the strongest compositional response occurs in the B-site/oxygen sublattice rather than through a major change in the Sr oxidation state.

The Fe 2p envelopes (Figure 6C) are asymmetric and contain the multiplet/satellite structure expected for mixed-valence ferrites, supporting coexistence of Fe^3+^ and Fe^4+^. Ce substitution changes the relative contribution of these components, demonstrating that B-site Ce perturbs the Fe redox equilibrium rather than acting as a chemically inert diluent. This redistribution is central to the transport response because electronic conduction in SrFeO_3−δ_ is associated with the Fe-derived network and is highly sensitive to oxygen deficiency and Fe-O-Fe geometry [20].

The O 1s spectra (Figure 6D) provide the clearest evidence for a non-monotonic defect response. The low-binding-energy component near 529–530 eV corresponds to lattice O^2−^, whereas the ~531 eV contribution is associated with defect-related/chemisorbed oxygen stabilized at undercoordinated surface sites; the higher-binding-energy tail is mainly associated with hydroxylated, carbonate-containing, or weakly adsorbed oxygen species. The relative defect-associated contribution increases with Ce incorporation and is most pronounced for x = 0.15, followed by a weaker response at x = 0.20. The ~531 eV component should not be equated quantitatively with vacancy concentration, but its evolution indicates that the x = 0.15 surface provides the most favorable population of defect-coupled oxygen species.

The Ce 3d region was also examined for the Ce-substituted SrFe_1−x_Ce_x_O_3−δ_ samples. However, reliable quantitative deconvolution of the Ce 3d envelope was not attempted because of the relatively low signal intensity and the intrinsically complex multiplet/satellite structure associated with Ce 3d photoemission. A similar limitation has been reported for Ce-doped SrFeO_3_-related perovskites, where Ce was detected by XPS but detailed Ce 3d analysis was considered unreliable owing to the weak signal and spectral complexity [21]. Therefore, the influence of Ce substitution on the surface redox environment was evaluated primarily from the Fe 2p and O 1s spectra. Changes in the Fe^3+^/Fe^4+^ balance and the relative contributions of lattice and surface oxygen species provide complementary evidence for Ce-induced modification of the redox state and oxygen-defect chemistry.

### 3.5. Conductivity Measurement

**Figure 7:**
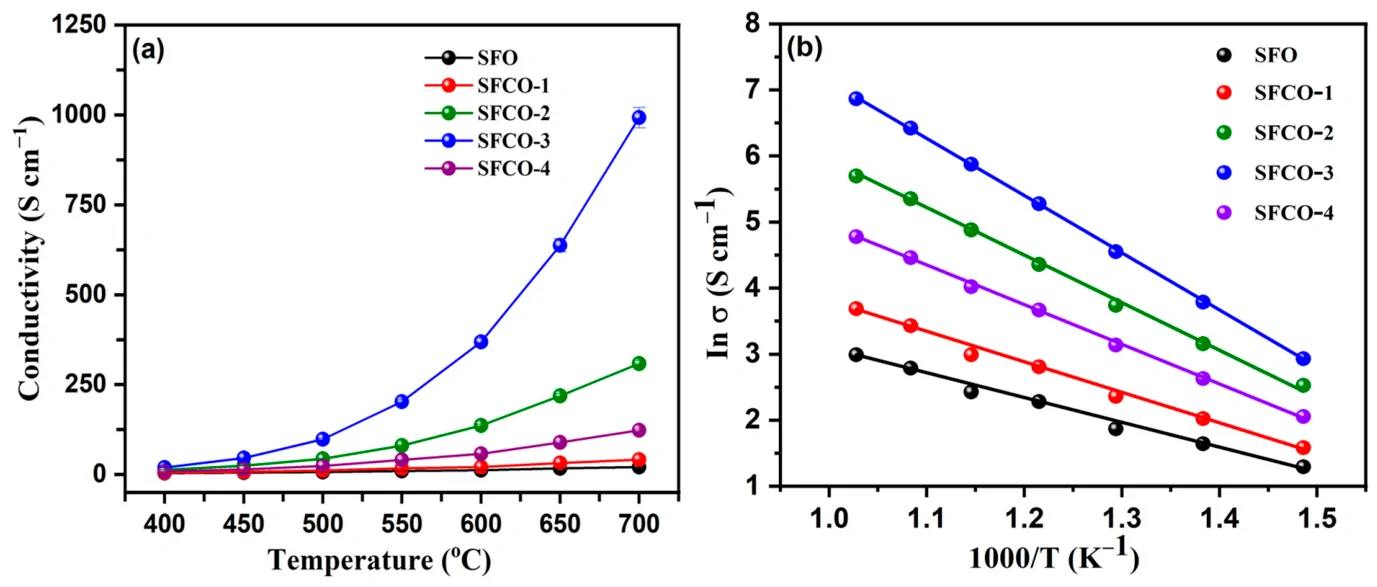
Electrical transport properties of SFO, SFCO-1, SFCO-2, SFCO-3, and SFCO-4: (**a**) electrical conductivity from 400 to 700 °C and (**b**) corresponding Arrhenius plots of ln σ versus 1000/T used to determine the apparent activation energies.

The temperature-dependent electrical conductivity of the SFO, SFCO-1, SFCO-2, SFCO-3 and SFCO-4 series is presented in Figure 7a. All compositions exhibit a continuous increase in conductivity between 400 and 700 °C, confirming thermally activated electrical transport. The magnitude of the conductivity, however, is strongly dependent on Ce content, as summarized in Table 2. At 700 °C, pristine SFO exhibits a conductivity of 19.91 S cm^−1^, which increases to 39.91 S cm^−1^ for x = 0.05 and then rises sharply to 298.21 S cm^−1^ for x = 0.10. The highest conductivity is obtained for SFCO-3, reaching 959.77 S cm^−1^ at 700 °C. Further increasing the Ce content to x = 0.20 causes the conductivity to decrease markedly to 118.66 S cm^−1^. The compositional response is therefore distinctly non-monotonic, with an optimum electrical transport response at x = 0.15. Electrical conduction in oxygen-deficient strontium ferrites is known to be highly sensitive to oxygen non-stoichiometry, Fe oxidation state, carrier concentration, carrier mobility, and the connectivity of the Fe-O-Fe framework [12,19].

The enhancement caused by moderate Ce incorporation is unlikely to originate from a single defect parameter. Substitution at the Fe site modifies the local coordination environment, oxygen-vacancy population, and Fe^3+^/Fe^4+^ distribution, all of which can influence the concentration and mobility of electronic charge carriers. In ferrite perovskites, mixed Fe valence is particularly important because electronic transport is associated with charge transfer between neighboring Fe sites through the Fe-O-Fe network [22,23,24]. In the present system, the pronounced increase in conductivity up to x = 0.15 is therefore consistent with a favorable balance between defect formation and preservation of an interconnected electronically active perovskite framework. This interpretation agrees with the accompanying XPS observations, which indicate pronounced defect-associated oxygen contributions together with mixed Fe oxidation states, as well as the XRD and HRTEM/SAED results showing retention of the principal perovskite structure.

The Arrhenius plots of ln σ versus 1000/T are shown in Figure 7b. Approximately linear relationships are observed over the investigated temperature range, indicating that the measured conductivity can be described by an apparent thermally activated process according to

(1) $$\sigma =\sigma_{0}\exp-\frac{E_{a}}{k_{B}T}$$

where, σ is the pre-exponential factor, E_a_ is the apparent activation energy, k_B_ is the Boltzmann constant, and T is the absolute temperature. The calculated activation energies are 0.320, 0.394, 0.610, 0.747, and 0.516 eV for (x = 0, 0.05, 0.10, 0.15, and 0.20), respectively. Thermally activated conductivity and composition-dependent activation energies have also been reported for substituted SrFeO_3−δ_-based oxides, where changes in oxygen stoichiometry, Fe valence, and B-site chemistry substantially alter the transport process [22,23,24].

A notable feature is that the x = 0.15 composition combines the highest absolute conductivity with the largest apparent activation energy, E_a_ = 0.747 eV. This does not represent a contradiction. Conductivity is determined not only by the activation barrier but also by the pre-exponential factor σ_0_, which contains contributions from the density of mobile carriers, the number of available transport sites, attempt frequency, and structural connectivity. Consequently, the comparatively high conductivity of SFCO-3 indicates that favorable carrier concentration and transport-path connectivity compensate for its higher apparent activation barrier. Studies on SrFeO_3−δ_ and related B-site-substituted ferrites similarly demonstrate that carrier mobility and conductivity can change substantially with oxygen stoichiometry and Fe-O-Fe connectivity, even when the apparent activation energy follows a different compositional trend [23,24].

At x = 0.20, the conductivity decreases sharply from 959.77 to 118.66 S cm^−1^ at 700 °C, although the activation energy drops from 0.747 to 0.516 eV, showing that a lower E_a_ alone does not ensure higher conductivity. Excessive Ce substitution can reduce the continuity of Fe-O-Fe transport pathways and alter the Fe^3+^/Fe^4+^ balance, as reported for heavily B-site-substituted strontium ferrites [6,23]. The decline may also be associated with the increased presence of minor CeO_2_/Sr_3_Fe_2_O_7−δ_ phases at higher Ce loading. Overall, SFCO-3 exhibits the highest conductivity in the series, reaching 959.77 S cm^−1^ at 700 °C, about 48 times that of pristine SrFeO_3−δ_. Therefore, SFCO-3 was selected for single-cell fabrication based on its superior overall transport behavior together with favorable defect chemistry and structural stability.

### 3.6. Electrochemical Characterization of the Asymmetric Cell

**Figure 8:**
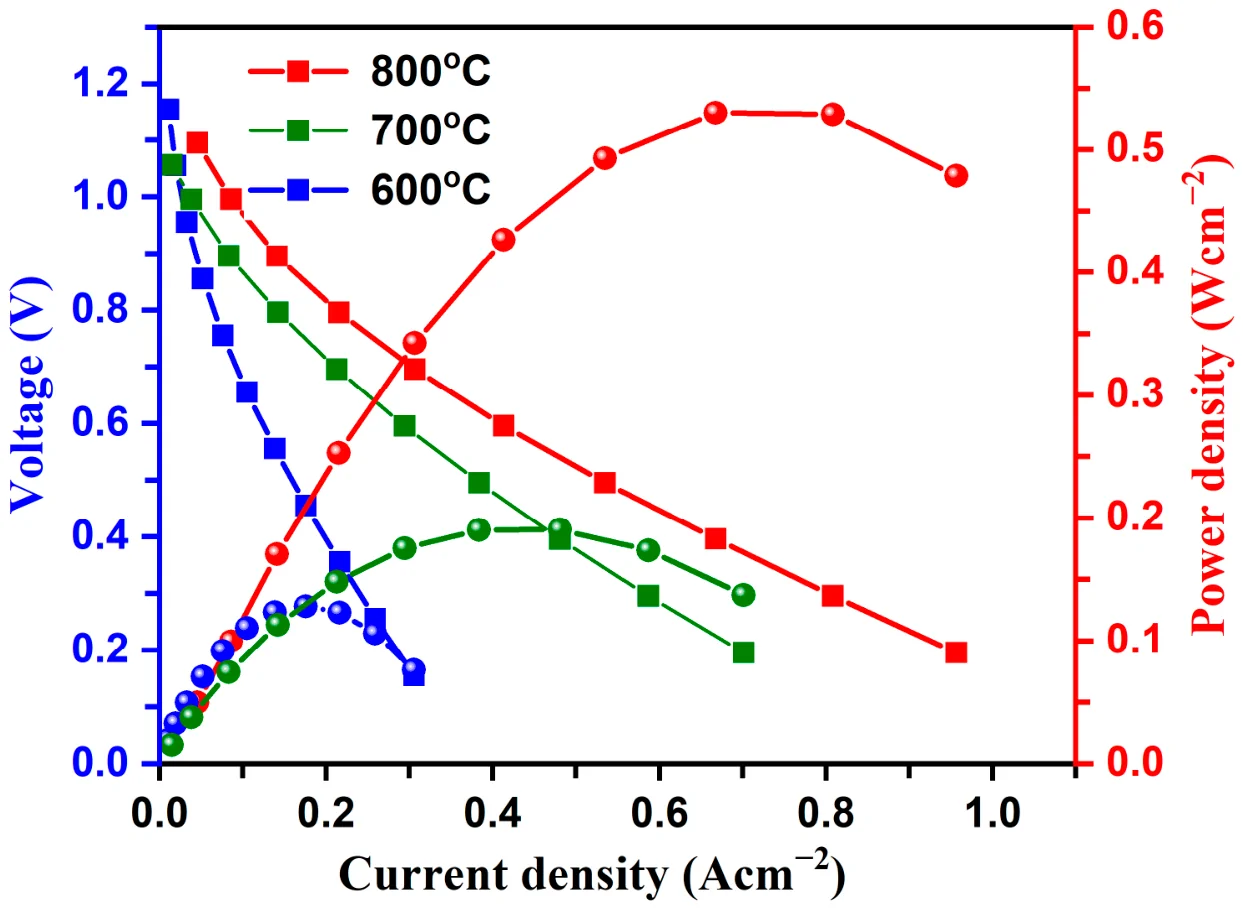
Current density-voltage (I-V) and corresponding power-density curves of the single cell employing SFCO-3 as the oxygen electrode at 600, 700, and 800 °C.

Figure 8 shows a plot of power density and cell voltage as a function of current density for the manufactured SOFC button cell of NiO-YSZ||YSZ||SrFe_0.85_Ce_0.15_O_3−δ_ cells with 1 cm^2^ active area and using H_2_ as a fuel and O_2_ as an oxidant in the temperature range of 600–800 °C. The cell has a high open circuit voltage (OCV) of 1.1 V at 800 °C. The I-V and I-P characteristics of the cell employing SFCO-3 as the oxygen electrode show a strong temperature dependence (Figure 8). Increasing the operating temperature reduces the voltage loss at a given current density and shifts the power-density maximum toward higher current, culminating in a peak power density of 0.53 W cm^−2^ at 800 °C. The response reflects the simultaneous acceleration of oxygen-electrode kinetics and oxide-ion transport through the cell as thermal activation increases. The absence of a sharp terminal voltage collapse over most of the measured range suggests that the dominant limitation is electrochemical/ohmic polarization rather than catastrophic gas-transport depletion under the present test conditions.

The measured output is competitive with several SrFeO_3−δ_ -derived cells, although direct ranking must be made cautiously because electrolyte thickness, cell architecture, electrode loading, gas composition, and active area differ among reports. Undoped SrFeO_3−δ_ delivered 202 mW cm^−2^ at 800 °C [5], while SrFe_0.9_Ti_0.1_O_3−δ_ reached 509 mW cm^−2^ under an electrolyte-supported symmetric configuration [8]. W-stabilized SrFe_0.8_W_0.2_O_3−δ_ reached 931 mW cm^−2^ at 850 °C with an LSGM electrolyte [7]. A-site Ce_0.2_Sr_0.8_FeO_3_ produced 482 mW cm^−2^ at 800 °C, increasing to 846 mW cm^−2^ after Ru modification [13]. Against this background, the 530 mW cm^−2^ output of the present B-site Ce composition is notable because it is achieved without Co and without a noble-metal co-catalyst. The result therefore supports B-site Ce as a viable route for improving the practical response of SrFeO_3−δ_ while retaining a chemically simple ferrite framework. The single-cell performance of the optimized SFCO-3 oxygen electrode is compared with representative SrFeO_3−δ_ -based cathode materials reported in the literature, as summarized in Table 3.

### 3.7. Impedance Response and Temperature-Dependent ORR Polarization

**Figure 9:**
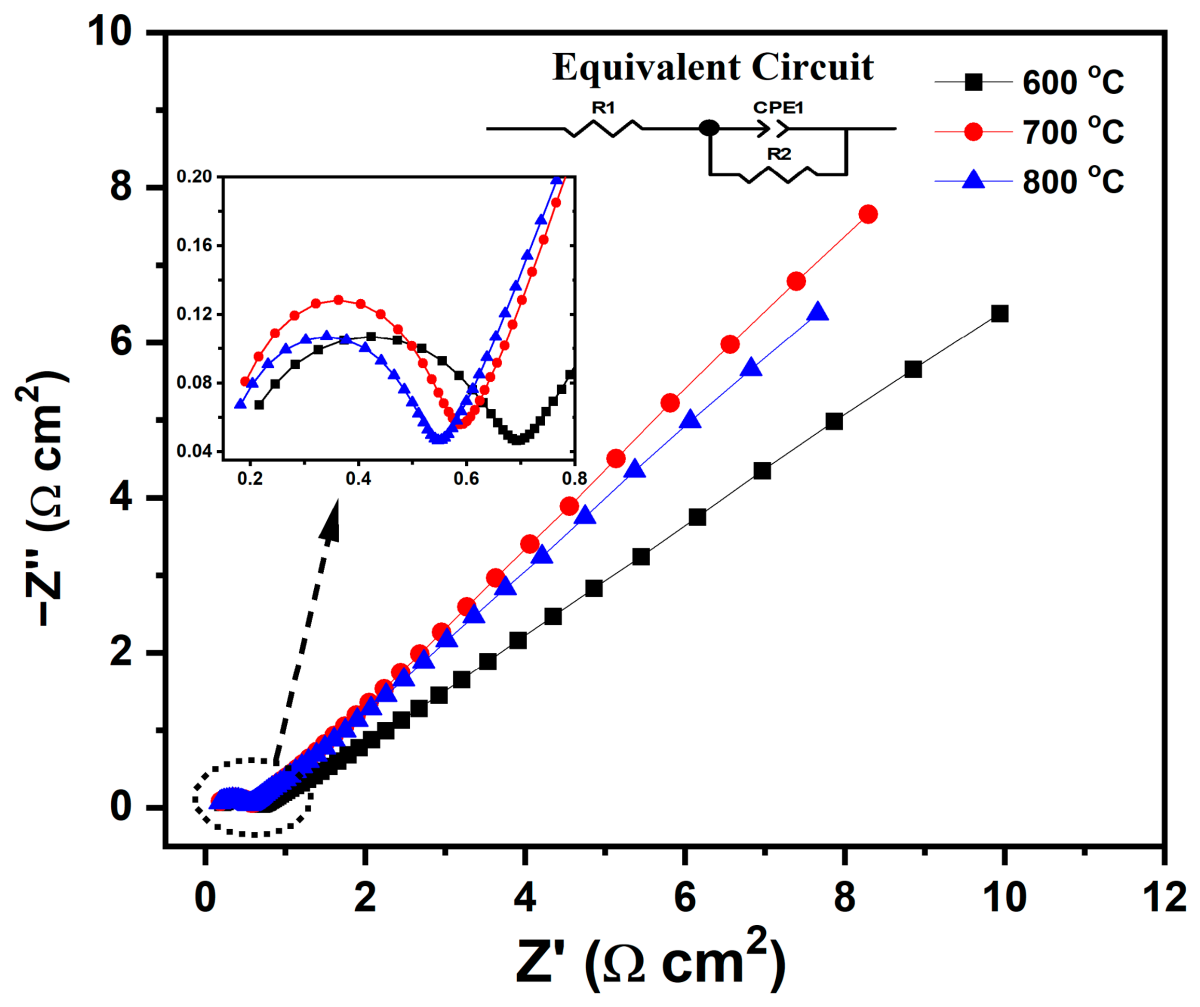
Nyquist impedance spectra of the single cell employing SFCO-3 as the oxygen electrode, measured at 600, 700, and 800 °C.

The Nyquist spectra of the SFCO-3 cell contract markedly with increasing temperature (Figure 9), demonstrating that both series resistance and electrode polarization are thermally activated. The fitted impedance parameters of the SFCO-3-based cell at 600–800 °C are summarized in Table 4. The fitted (R_s_) decreases from 0.21 Ω at 600 °C to 0.19 Ω at 700 °C and 0.16 Ω at 800 °C, corresponding to a 23.8% reduction over the full temperature interval. This trend is consistent with enhanced ionic conduction through the electrolyte and lower contact/interfacial resistance at elevated temperature.

The dominant change occurs in the electrode arc. Within the single-arc representation used here, (R_ct_) is equivalent to the polarization resistance (R_p_) and decreases from 0.47 Ω at 600 °C to 0.40 Ω at 700 °C and 0.36 Ω at 800 °C. Thus, (R_p_) falls by 23.4% between 600 and 800 °C, with the largest decrease occurring between 600 and 700 °C. The full-cell area-specific polarization resistance, calculated as R_p_A using the 1 cm^2^ active area, decreased from 0.47 Ω cm^2^ at 600 °C to 0.36 Ω cm^2^ at 800 °C. The depressed, non-ideal arc indicates a distribution of relaxation processes rather than a single elementary step, so the full arc should not be assigned exclusively to elementary electron transfer. For SrFeO_3−δ_-derived MIEC electrodes, the polarization response generally contains coupled contributions from oxygen adsorption/dissociation, surface exchange, interfacial charge transfer, oxygen incorporation, and solid-state transport. DRT analysis of SrFe_0.8_W_0.2_O_3−δ_ has specifically identified dissociation of adsorbed oxygen and charge transfer as major cathodic limitations [7].

The total resistance correspondingly decreases from 0.68 Ω at 600 °C to 0.59 Ω at 700 °C and 0.52 Ω at 800 °C, giving an overall reduction of 23.5%. (R_p_) accounts for approximately 68–69% of (R_total_) at all three temperatures, showing that electrode kinetics remain the dominant contribution to the measured cell loss, although the ohmic component is not negligible. This finding provides the quantitative link between impedance and the I-V/I-P response: the higher power density at 800 °C is principally enabled by suppression of oxygen-electrode polarization. The result also reinforces the structure-defect interpretation established above. SFCO-3 sample combines a defect-active surface with a still-connected Fe-derived electronic network; increasing temperature then activates oxygen exchange/incorporation and transport through this optimized framework, producing the observed decrease in (R_p_).

Ce substitution initially expands the SrFeO_3−δ_ lattice, but further substitution produces little additional expansion, and weak secondary-phase reflections appear at higher Ce contents, suggesting a limit to Ce incorporation under the present synthesis conditions. The accompanying changes in the surface Fe^3+^/Fe^4+^ ratio and O 1s components indicate a modified surface defect environment, although XPS does not quantify bulk oxygen vacancies. Conductivity reaches its maximum at x = 0.15 (SFCO-3) before declining at x = 0.20, consistent with a balance between Ce-induced changes in defect chemistry and preservation of Fe-O-Fe electronic pathways. SFCO-3 was selected for single-cell validation on the basis of these combined structural, surface-chemical, microstructural, and transport results. Its conductivity was highest despite its reported apparent porosity being the highest in the series, although porosity differences remain relevant when comparing pellets. The SFCO-3 cell delivered its highest measured peak power density of 0.53 W cm^−2^ at 800 °C, while its full-cell area-specific polarization resistance decreased from 0.47 Ω cm^2^ at 600 °C to 0.36 Ω cm^2^ at 800 °C. Thus, 800 °C gave the strongest performance within the tested range; long-term stability data are needed to identify a practical operating temperature. The peak power density exceeds the 202 mW cm^−2^ reported for undoped SrFeO_3−δ_ [5] and is close to the 509 mW cm^−2^ reported for SrFe_0.9_Ti_0.1_O_3−δ_ [8], although differences in cell architecture, electrolyte thickness, and test conditions limit direct comparison. Testing the other compositions in single cells would be necessary to establish whether SFCO-3 also gives the best cell-level performance across the series.

Taken together, the data define a non-monotonic role for B-site Ce in SrFeO_3−δ_. The first Ce addition expands the average lattice, showing that the B-site/oxygen sub lattice is strongly perturbed. Increasing Ce to x = 0.15 preserves the perovskite framework and nanoscale crystallinity while maximizing the defect-associated O 1s response and electrical conductivity. In this regime, Ce-induced structural relaxation and Fe-redox redistribution create a defect-active oxygen environment without excessively interrupting Fe-O-Fe electronic connectivity. The result is the best transport balance within the series and a correspondingly active oxygen electrode.

Beyond the optimum, the structural and transport trends diverge. The lattice parameter no longer increases, CeO_2_/Sr_3_Fe_2_O_7−δ_ contributions become more evident, particle-size heterogeneity increases, and the conductivity declines. These observations indicate that additional Ce is increasingly accommodated outside the electronically active perovskite network or introduces local configurations that no longer benefit charge transport. The x = 0.15 maximum should therefore be viewed as an optimum defect-carrier balance rather than a simple maximum in oxygen-vacancy concentration. This distinction is consistent with the broader ferrite literature, where B-site dopants can improve oxygen transport while simultaneously reducing electronic mobility when they dilute the transition-metal conduction network [6,12]. The present single-cell and EIS data demonstrate that this balance translates directly into ORR performance, linking composition to cell output through a coherent structure-defect-transport pathway.

## 4. Conclusions

B-site Ce substitution provides an effective but compositionally limited route for tuning cobalt-free SrFeO_3−δ_ oxygen electrodes. SrFe_1-x_Ce_x_O_3−δ_ (x = 0 to 0.20) retains the dominant perovskite framework, but the structural response is strongly non-linear: an initial lattice expansion is followed by lattice saturation and weak CeO_2_/Sr_3_Fe_2_O_7−δ_ segregation at higher Ce contents. XPS shows that Ce modifies the Fe^3+^/Fe^4+^ and surface oxygen environments, while the x = 0.15 composition exhibits the most favorable defect-associated oxygen response together with the highest electrical conductivity. HRTEM/SAED further confirms that this composition preserves well-crystallized perovskite domains. The SFCO-3-based cell reaches 0.53 W cm^−2^ at 800 °C, while its polarization resistance decreases from 0.47 Ω at 600 °C to 0.36 Ω at 800 °C. The combined results show that the performance maximum is governed by an optimum balance between Ce-induced defect activation and preservation of Fe-O-Fe electronic connectivity; excessive Ce instead promotes phase segregation and transport deterioration. B-site Ce engineering therefore offers a viable strategy for developing cobalt-free SrFeO_3-δ_ cathodes for intermediate-temperature SOFCs.

## Figures and Tables

**Table 1:** Rietveld-refined structural and profile parameters of SFO, SFCO-1, SFCO-2, SFCO-3 and SFCO-4.

Parameter	SFO	SFCO-1	SFCO-2	SFCO-3	SFCO-4
a = b = c (Å)	3.8617(13)	3.8758(8)	3.8745(15)	3.8746(15)	3.8745(15)
V (Å^3^)	57.59(2)	58.22(1)	58.16(2)	58.17(2)	58.16(2)
Calculated density (g cm^−3^)	5.52	5.46	5.46	5.46	5.46
Robs (%)	2.65	2.58	2.85	2.71	2.34
Rp, profile (%)	5.10	5.07	5.26	5.12	5.03
GOF	3.02	3.01	3.05	3.04	3.01

**Table 2:** Electrical conductivity at 700 °C and apparent activation energy (E_a_) of SFO, SFCO-1, SFCO-2, SFCO-3, and SFCO-4.

Composition	Conductivity at 700 °C (S cm^−1^)	Activation Energy E_a_ (eV)
SFO	19.91	0.32
SFCO-1	39.91	0.394
SFCO-2	298.21	0.61
SFCO-3	959.77	0.747
SFCO-4.	118.66	0.516

**Table 3:** Selected literature comparison of SrFeO_3−δ_-based single-cell performance. Values are not directly comparable without considering cell architecture and electrolyte thickness.

Oxygen Electrode	Temperature (°C)	Power Density (mW cm^−2^)	Reference
SrFeO_3−δ_	800	202	[5]
SrFe_0.9_Ti_0.1_O_3−δ_	800	509	[8]
SrFe_0.8_Wo_0.2_O_3−δ_	850	931	[7]
Ce_0.2_Sr_0.8_FeO_3−δ_ (A-site Ce)	800	482	[13]
SrFe_0.85_Ce_0.15_O_3−δ_	800	530	Present work

**Table 4:** Fitted electrochemical impedance parameters of the single cell employing SFCO-3 as the oxygen electrode at 600, 700, and 800 °C.

Temperature (°C)	R_s_ (Ω)	R_ct_ (Ω)	R_p_A (Ω)	R_total_ (Ω)
600	0.21	0.47	0.47	0.68
700	0.19	0.40	0.40	0.59
800	0.16	0.36	0.36	0.52

## Data Availability

The original contributions presented in this study are included in the article/Supplementary Material. Further inquiries can be directed to the corresponding authors.

## Supplementary Materials

The following supporting information can be downloaded at: https://www.mdpi.com/article/10.3390/ma19194245/s1, Figure S1: SEM-EDS characterization of (I) SFO, (II) SFCO-1, (III) SFCO-2, (IV) SFCO-3, and (V) SFCO-4. For each sample, (a) shows the SEM image with a magnified view of the marked region, (b) the EDS spectrum with an inset of the combined elemental map, and (c) the SEM reference image used for mapping. The individual maps show (d) Sr, (e) Ce, (f) Fe, and (g) O for SFCO-1to SFCO-4. For SFO, which contains no Ce, panels (d–f) show Sr, Fe, and O, respectively.
