# Supplementary material for: High-Performance Cobalt-Free SrFe1−xCexO3−δ Cathode Enabled by Ce-Driven Defect Engineering for IT-SOFCs
Source: Materials (Basel). 2026 Oct 7;19(19):4245. doi: 10.3390/ma19194245 (PMC13666004; doi:10.3390/ma19194245)
Supplement: Supplementary file 1 [file materials-19-04245-s001.zip › materials-4576538-supplementary.pdf]

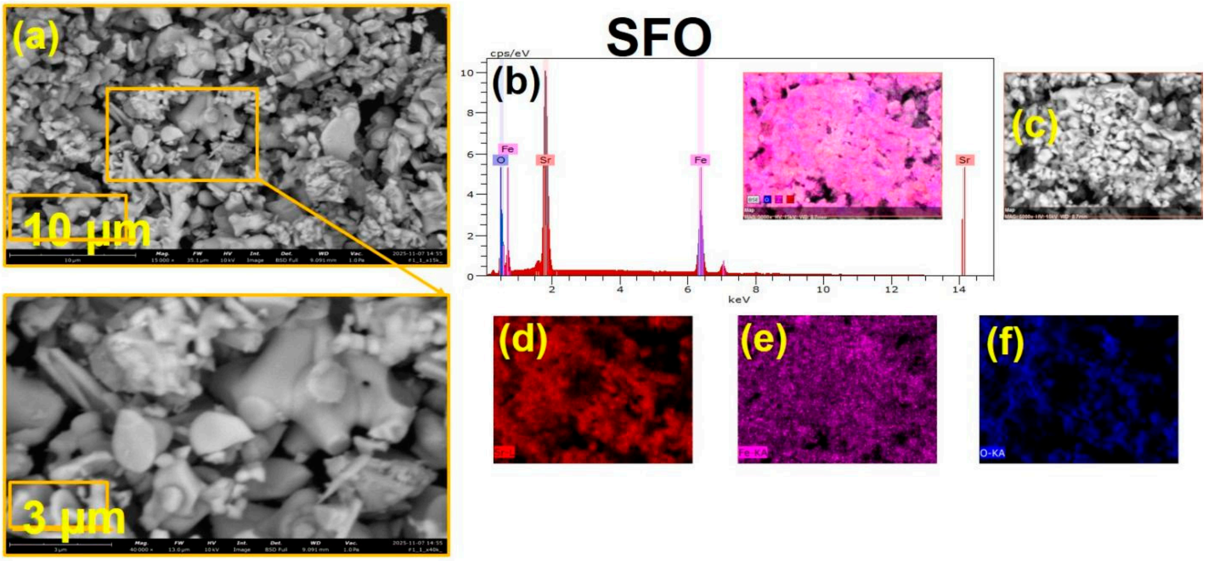
SFO
(a)
10 µm
3 µm
(b)
cps/eV
keV
O
Fe
Sr
(c)
(d)
(e)
(f)
O-KA

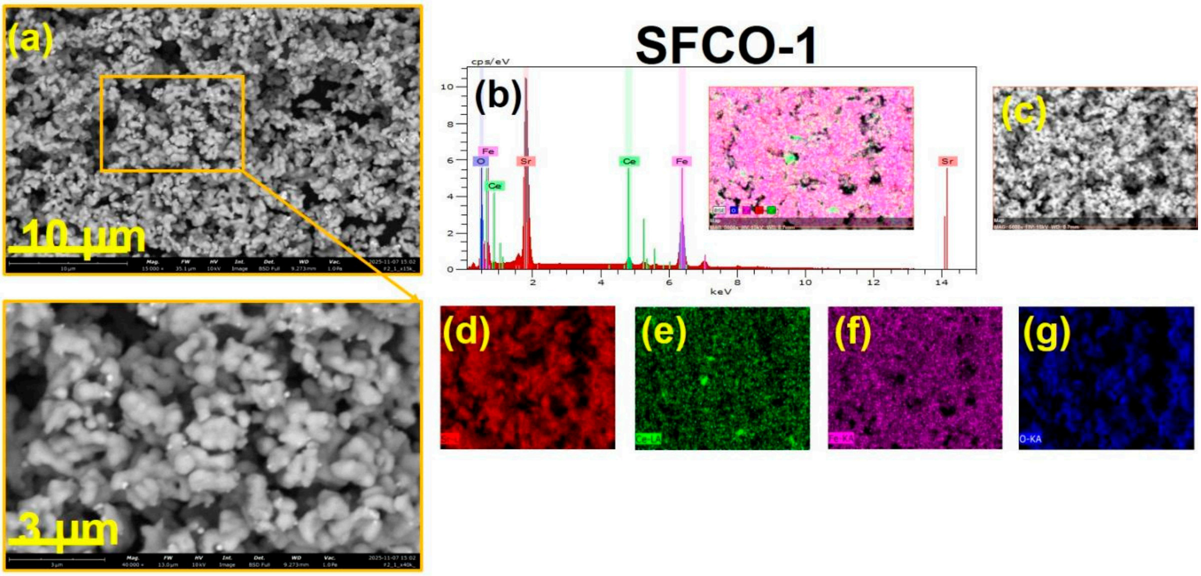
SFCO-1
(a)
10 µm
3 µm
(b)
cps/eV
keV
O
Fe
Ce
Sr
(c)
(d)
(e)
(f)
(g)
O-KA

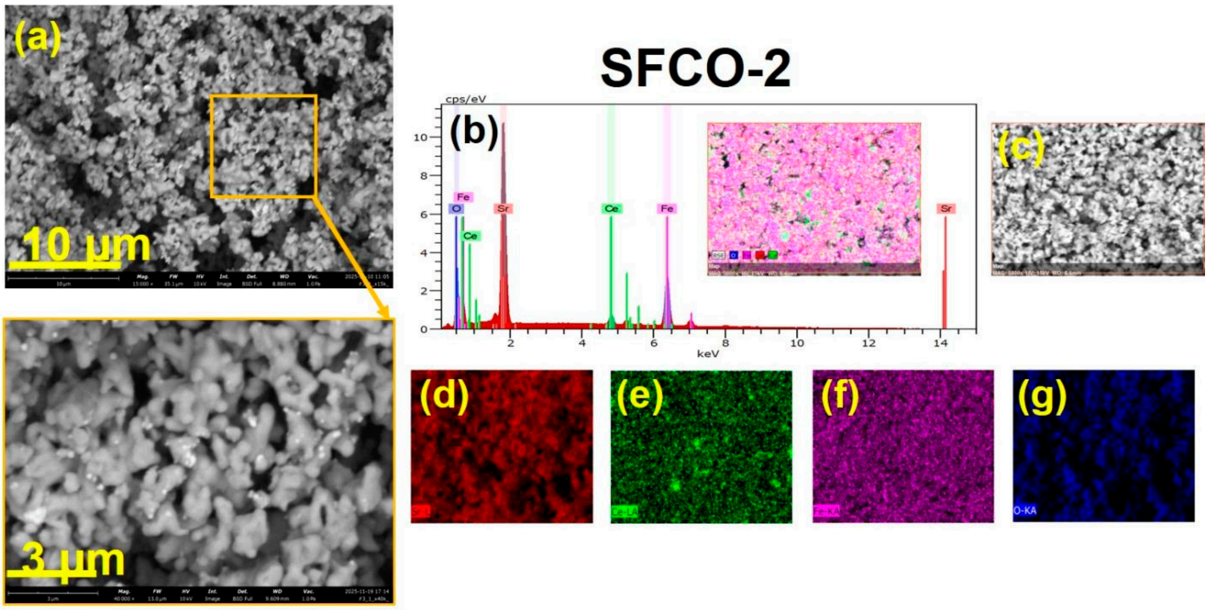

SFCO-2
(a)
10 µm
3 µm
(b)
cps/eV
O
Fe
Ce
Sr
keV
(c)
(d)
(e)
(f)
(g)
O-KA

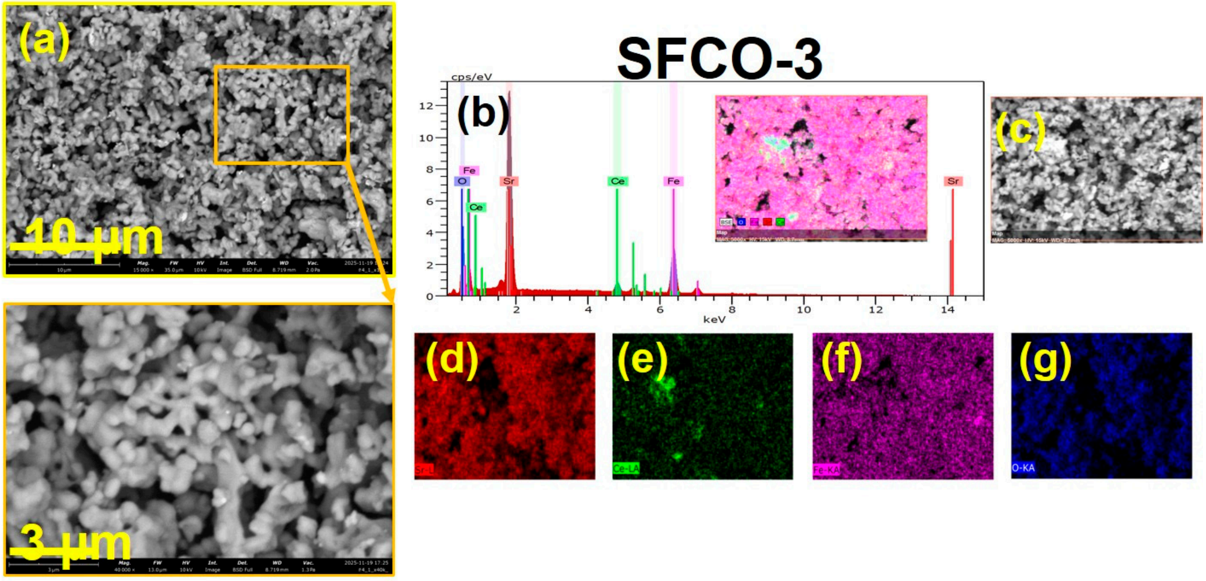

SFCO-3
(a)
10 µm
3 µm
(b)
cps/eV
O
Fe
Ce
Sr
keV
(c)
(d)
(e)
(f)
(g)
Ce-LA
O-KA

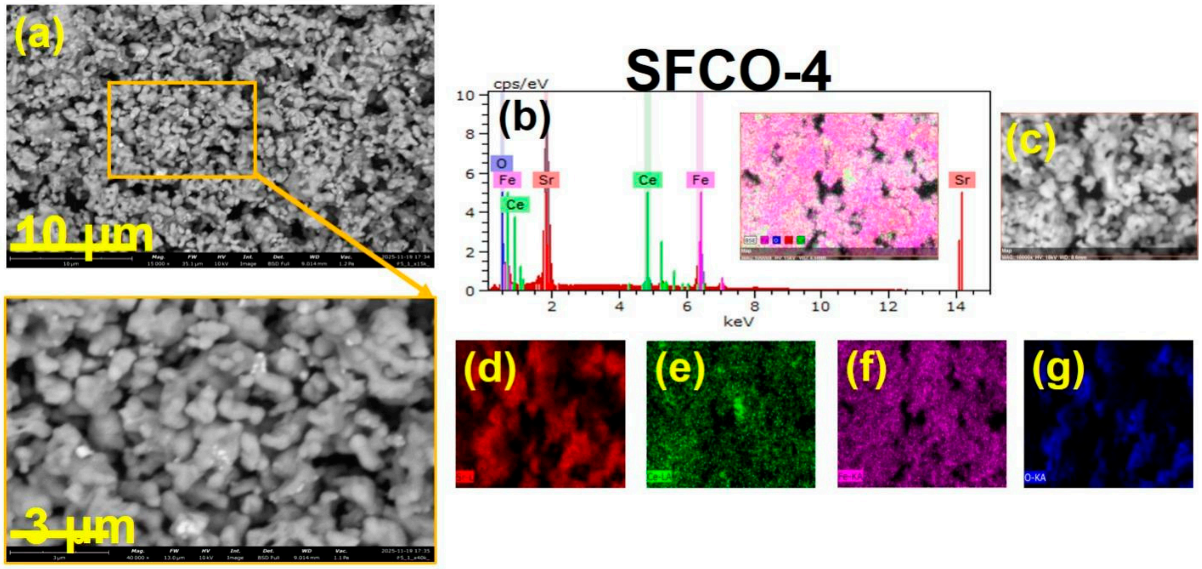

Supplementary Figure S1. SEM-EDS characterization of (I) SFO, (II) SFCO-1, (III) SFCO-2, (IV) SFCO-3, and (V) SFCO-4. For each sample, (a) shows the SEM image with a magnified view of the marked region, (b) the EDS spectrum with an inset of the combined elemental map, and (c) the SEM reference image used for mapping. The individual maps show (d) Sr, (e) Ce, (f) Fe, and (g) O for SFCO-1to SFCO-4. For SFO, which contains no Ce, panels (d-f) show Sr, Fe, and O, respectively.
